# The antiviral protein viperin regulates chondrogenic differentiation via CXCL10 protein secretion

**DOI:** 10.1074/jbc.RA119.007356

**Published:** 2019-02-04

**Authors:** Mandy M. F. Steinbusch, Marjolein M. J. Caron, Don A. M. Surtel, Guus G. H. van den Akker, Paul J. van Dijk, Franziska Friedrich, Bernhard Zabel, Lodewijk W. van Rhijn, Mandy J. Peffers, Tim J. M. Welting

**Affiliations:** From the ‡ Laboratory for Experimental Orthopedics, Department of Orthopedic Surgery and; the § Department of Anatomy and Embryology, Maastricht University, NL-6202 AZ Maastricht, The Netherlands,; the ¶ University Heart Centre Freiburg, Faculty of Medicine, University of Freiburg, Institute for Experimental Cardiovascular Medicine, 79110 Freiburg, Germany,; the ‖ Medical Faculty, Otto van Guericke University of Magdeburg, 39106 Magdeburg, Germany, and; the ** Department of Musculoskeletal Biology, Institute of Ageing and Chronic Disease, University of Liverpool, Liverpool L69 3BX, United Kingdom

**Keywords:** cartilage biology, chondrogenesis, small nucleolar RNA (snoRNA), transforming growth factor beta (TGF-B), chemokine, secretion, C-X-C motif chemokine ligand 10, cartilage-hair hypoplasia, chondrogenic differentiation, RMRP snoRNA, viperin

## Abstract

Viperin (also known as radical SAM domain–containing 2 (RSAD2)) is an interferon-inducible and evolutionary conserved protein that participates in the cell's innate immune response against a number of viruses. *Viperin* mRNA is a substrate for endoribonucleolytic cleavage by RNase mitochondrial RNA processing (MRP) and mutations in the RNase MRP small nucleolar RNA (snoRNA) subunit of the RNase MRP complex cause cartilage-hair hypoplasia (CHH), a human developmental condition characterized by metaphyseal chondrodysplasia and severe dwarfism. It is unknown how CHH-pathogenic mutations in RNase MRP snoRNA interfere with skeletal development, and aberrant processing of RNase MRP substrate RNAs is thought to be involved. We hypothesized that viperin plays a role in chondrogenic differentiation. Using immunohistochemistry, real-time quantitative PCR, immunoblotting, ELISA, siRNA-mediated gene silencing, plasmid-mediated gene overexpression, label-free MS proteomics, and promoter reporter bioluminescence assays, we discovered here that viperin is expressed in differentiating chondrocytic cells and regulates their protein secretion and the outcome of chondrogenic differentiation by influencing transforming growth factor β (TGF-β)/SMAD family 2/3 (SMAD2/3) activity via C-*X*-C motif chemokine ligand 10 (CXCL10). Of note, we observed disturbances in this viperin–CXCL10–TGF-β/SMAD2/3 axis in CHH chondrocytic cells. Our results indicate that the antiviral protein viperin controls chondrogenic differentiation by influencing secretion of soluble proteins and identify a molecular route that may explain impaired chondrogenic differentiation of cells from individuals with CHH.

## Introduction

Viperin, also known as RSAD2, is an interferon-inducible and evolutionary conserved protein that participates in the cell's innate immune response against a number of viruses. Viperin localizes to the cytosolic face of the endoplasmic reticulum (ER),^3^ mitochondria, and lipid droplets. It is described to exert its antiviral properties via several pathways, including inhibition of soluble protein secretion, alterations of mitochondrial energy metabolism, inhibition of virus replication in lipid droplets, and modulation of cellular signaling events (1). The mRNA of viperin was identified as a substrate for endoribonucleolytic cleavage by the RNase MRP small nucleolar ribonucleoprotein complex (2). Mutations in the RMRP gene, which encodes the essential small nucleolar (snoRNA) subunit of the RNase MRP complex, are known to be the cause of the human genetic disease cartilage-hair hypoplasia (CHH; Online Mendelian Inheritance in Man accession no. 250250) (3, 4). A major phenotypic hallmark of CHH is impaired skeletal development characterized by metaphyseal chondrodysplasia, leading to a severe form of dwarfism (5). Growth plates are central drivers in the formation of the skeleton, and their endochondral ossification depends on a tightly orchestrated continuing process of chondrogenic differentiation, ultimately resulting in longitudinal growth of the long bones (6). Although a number of RNase MRP substrates have been identified in man (2, 4, 7–9), including viperin, there is a major lack of studies investigating the role of RNase MRP substrate RNAs in development and in chondrogenic differentiation in particular. It is expected that insight into the involvement of RNase MRP substrates in chondrogenic differentiation will provide insight into the molecular mechanism leading to impaired skeletal development in CHH. In concert with viperin mRNA as a substrate for RNase MRP cleavage, viperin expression levels have been described to be increased after knockdown of protein components of the RNase MRP complex in HEp-2 cells (2), in leukocytes of CHH patients (10), and recently we demonstrated that viperin expression is also up-regulated after knockdown of RMRP RNA in chondrogenic differentiating ATDC5 cells (11). Because increased viperin expression as a result of interference with RNase MRP function appears to be conserved among several cell types, we hypothesized that viperin regulates the course of chondrogenic differentiation. In this study we examined the expression of viperin, determined its role in chondrocyte protein secretion, and investigated its cross-talk with the TGF-β/SMAD2/3 signaling pathway in chondrogenic differentiation. We discovered that viperin is expressed in differentiating chondrocytic cells, regulates their protein secretion, and impacts the outcome of the chondrogenic differentiation program through influencing TGF-β/SMAD2/3 activity via CXCL10. Our data show for the first time that the antiviral protein viperin regulates chondrogenic differentiation by influencing the secretion of soluble proteins and highlights its involvement in impaired chondrogenic differentiation in CHH patient cells.

## Results

### Viperin protein is expressed throughout chondrocyte differentiation

**Figure 1.**
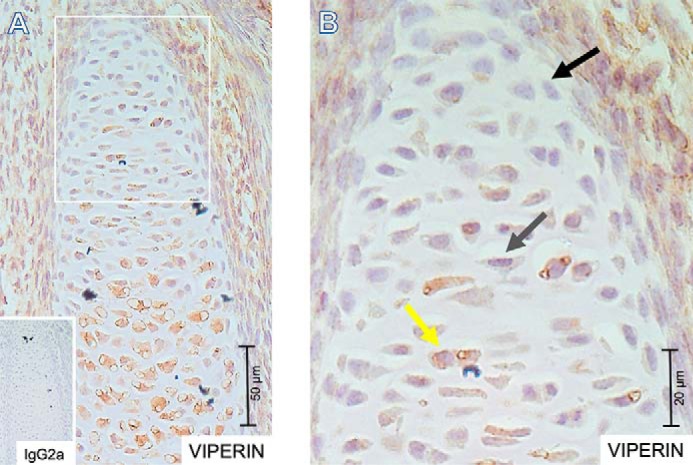
**Viperin expression in the developing embryonal growth plate.** 5-μm-thick formalin-fixed paraffin-embedded tissue sections were prepared from embryonic day 15.5 NMRI mouse embryos. Spatiotemporal expression of viperin was detected immunohistochemically. IgG2a was used as an isotype control. *A*, overview of a representative immunohistochemically stained growth plate with IgG2a negative control in the *inset. B*, indicated area from *A* enlarged. The *black arrow* indicates a representative cell with barely detectable viperin expression; the *gray arrow* indicates a representative cell with weak viperin expression; and the *yellow arrow* indicates a representative cell with high viperin expression. The *scale bars* are indicated for magnification reference.

To investigate whether viperin is expressed during early chondrogenic differentiation, tissue sections were prepared from embryonic day 15.5 mouse embryos. Expression of viperin was detected in growth plate chondrocytes throughout the developing growth plate, with the highest expression levels in proliferating and terminally differentiating hypertrophic chondrocytes (Fig. 1*A*). Cells in the upper zone of the growth plate displayed dispersed viperin positivity; viperin expression was either barely detectable in individual cells, was weakly expressed, or was relatively high expressed in individual cells (Fig. 1*B*).

**Figure 2.**
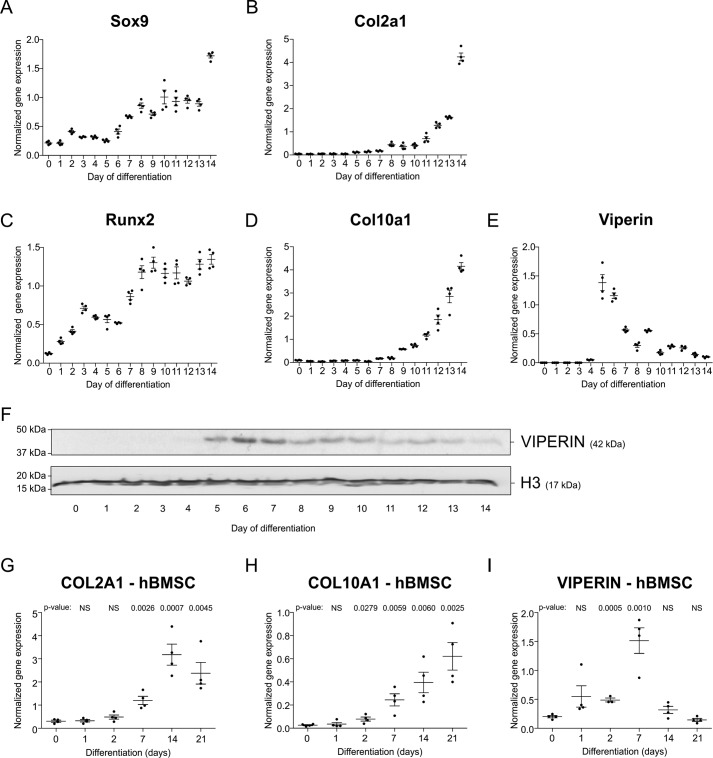
**Viperin is expressed during chondrogenic differentiation of ATDC5 and hBMSCs.** ATDC5 cells were differentiated in the chondrogenic lineage for 14 days. Different stages of chondrogenic differentiation were confirmed by measuring mRNA expression of Sox9 (*A*), Col2a1 (*B*), Runx2 (*C*), and Col10a1 (*D*) by RT-qPCR. Expression of viperin mRNA during chondrogenic differentiation was determined by RT-qPCR (*E*) and immunoblotting (*F*). Gene expression data (*A–E)* was normalized to β-actin mRNA levels, and individual normalized values are presented in dot plots. For Viperin immunoblotting (*F*), Histone 3 (H3) was used as a loading control. Primary hBMSCs of four human subjects were differentiated in the chondrogenic lineage for 1, 2, 7, 14, and 21 days. Chondrogenic differentiation was confirmed by measuring COL2A1 and COL10A1 mRNA expression (*G* and *H*). In same samples, expression of VIPERIN mRNA was measured (*I*). hBMSC gene expression data (*G* and *H*) was normalized to CYCLOPHILIN mRNA levels. Individual data points in dot plots represent the average values of four biological replicates of one hBMSC donor. The *p* values are indicated. *NS*, not significant. The presented graphs are representative examples of three independent experiments. *Error bars* represent means ± S.E.

We further interrogated potential viperin expression during chondrogenic differentiation of the ATDC5 cell line (12, 13) and of primary human bone marrow stem cells (hBMSCs) (14). Chondrogenic differentiation of ATDC5 follows a well-defined cellular differentiation program with temporally increasing Sox9 and Col2a1 levels (Fig. 2, *A* and *B*) and increasing levels of Runx2 and Col10a1 (Fig. 2, *C* and *D*). During the first 3 days of ATDC5 chondrogenic differentiation, viperin mRNA expression was hardly detectable (Fig. 2*E*). From day 4 through day 14 (the latest time point tested), viperin mRNA expression was induced as compared with undifferentiated ATDC5 cells (*t* = 0) and with a prominent peak expression at days 5 and 6 in differentiation. Similar expression dynamics were observed for viperin protein expression in these chondrogenic cultures (Fig. 2*F*). During chondrogenic differentiation of hBMSCs (COL2A1 and COL10A1 mRNA expression in Fig. 2, *G* and *H*), peak induction of viperin mRNA expression was detected at day 7 of differentiation (Fig. 2*I*), which is in agreement with viperin expression during ATDC5 chondrogenic differentiation. Collectively these data show that developing chondrocytes express viperin *in vivo* and *in vitro*, with undetectable to low expression in the early differentiation phase and a profoundly increased expression later in differentiation.

### Viperin knockdown reduces whereas viperin overexpression increases protein secretion

**Figure 3.**
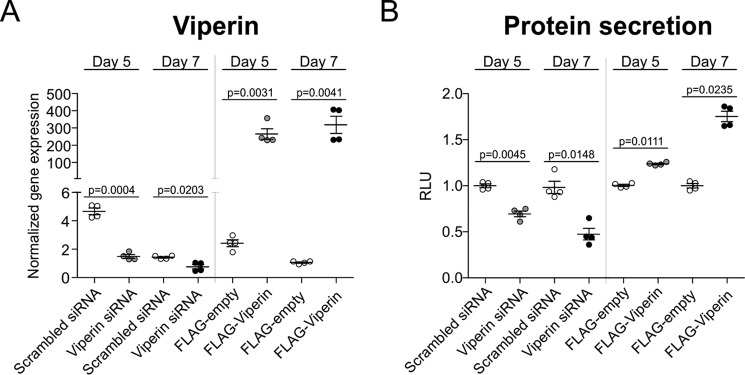
**Viperin knockdown reduces, whereas viperin overexpression increases overall protein secretion.** A CMV promoter-driven secreted Gaussia luciferase plasmid (pGluc-CMV) was co-transfected with a CMV promoter-driven firefly luciferase pGL4.20-CMV on day 3 or 5 of ATDC5 chondrogenic differentiation. Then on day 4 or 6, viperin expression was either reduced by transfection of a viperin-specific siRNA duplex (Viperin siRNA) or a scrambled control siRNA duplex (Scrambled siRNA); or viperin expression was further induced by transfection of a p3xFLAG-viperin plasmid (FLAG-Viperin) or the empty p3xFLAG plasmid (FLAG-empty). Reduced or induced viperin expression (normalized to β-actin) was confirmed by RT-qPCR at day 5 or 7 in differentiation (*A*) and individually presented in dot plots. Bioluminescence was assessed at day 5 or 7 in differentiation in culture supernatant (Gaussia luciferase) and cells (firefly luciferase). Gaussia bioluminescence was normalized to the firefly signal. Normalized relative light units (RLU) of controls were set at 1, and condition RLUs were calculated relative to the control RLUs and individually presented in dot plots (*B*). For statistical evaluation, an independent samples *t* test was performed relative to the corresponding controls using GraphPad Prism 5. Data are presented of four biological replicates. The *p* values are indicated and *error bars* indicate mean ± S.E. Presented graphs are examples of three individual experiments.

Previous work by Hinson and Cresswell (15) showed that viperin regulates protein secretion from the ER, and it has been suggested that this is one of the mechanisms by which viperin confers its cellular antiviral activity. Because there is no other cell biological role identified for viperin so far that could explain its expression in differentiating chondrocytes, we postulated that viperin regulates protein secretion in differentiating chondrocytes. To investigate total protein secretion in relation to viperin levels, we introduced a secretable Gaussia-luciferase (pGluc-CMV) at either day 3 or 5 in ATDC5 differentiation, followed by transfection of either a viperin siRNA duplex or a p3xFLAG-viperin plasmid at day 4 or 6 in differentiation, followed by sampling at day 5 or 7, respectively. Reduced expression of viperin at day 5 or 7 in differentiation was demonstrated upon siRNA-mediated knockdown (Fig. 3*A*), and ectopic expression of FLAG-viperin was confirmed at the same time points (Fig. 3*A*). Bioluminescent analyses showed that the amount of secreted Gaussia-luciferase was reduced following viperin knockdown and increased with viperin overexpression (Fig. 3*B*). This was the case at both days 5 and 7 of differentiation. These data indicate that overall cellular protein secretion responds to changing viperin levels during chondrogenic differentiation.

### The viperin-controlled secretome influences chondrogenic differentiation

**Figure 4.**
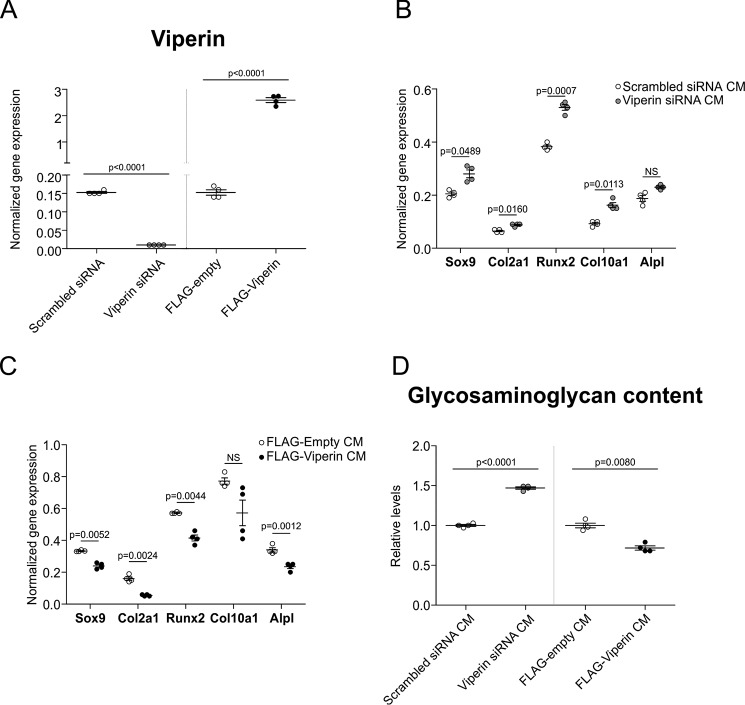
**The viperin secretome influences chondrogenic differentiation.** At day 5 in ATDC5 chondrogenic differentiation, viperin expression was either reduced by transfection of a viperin-specific siRNA duplex (Viperin siRNA) or a scrambled control siRNA duplex (Scrambled siRNA); or viperin expression was further induced by transfection of a p3xFLAG-viperin plasmid (FLAG-Viperin) or the empty p3xFLAG plasmid (FLAG-empty). The cells were then further differentiated until day 7, and viperin expression levels were determined (*A*). Conditioned culture supernatant (CM) from these donor cultures was harvested at day 7, and newly seeded ATDC5 cells were then differentiated with the CM supernatants for 7 days. RNA was isolated and Sox9, Col2a1, Runx2, Col10a1, and Alpl gene expression was determined in these samples (*B* and *C*). Glycosaminoglycan content (Alcian blue assay) was determined in ATDC5 cultures differentiated in CM supernatant from donor cultures in which viperin expression was reduced or further induced (*D*). RT-qPCR data were normalized to β-actin mRNA levels, and individual normalized values are presented as dot plots. Glycosaminoglycan content data are presented as fold change relative to the corresponding control condition. For statistical evaluation an independent samples *t* test was performed relative to the corresponding control condition using GraphPad Prism 5. The *p* values are indicated. *NS*, not significant. All presented data were acquired from four biological replicates, and *error bars* indicate means ± S.E. Graphs are representative examples of three individual experiments.

Considering the central role secreted signaling molecules play during chondrogenic differentiating (6, 16), we next questioned whether the observed viperin-controlled protein secretion plays a role in determining the chondrogenic outcome of the differentiation process via control over the chondrocyte's secretome. We investigated this possibility by reducing endogenous viperin levels or overexpressing viperin in differentiating ATDC5 chondrocytes and collecting conditioned media (CMs) from these donor cultures. These CMs were subsequently used to differentiate new ATDC5 cultures. Altered chondrogenic capacity of these cultures would reveal whether reduced or increased viperin levels during chondrogenic differentiation differentially influence the chondrocyte's secretome with downstream consequences for chondrogenic differentiation. Knockdown or overexpression of viperin was confirmed in the donor cultures from which CM were obtained (Fig. 4*A*). Differentiation of ATDC5 cells in CM obtained from donor cultures in which viperin levels were reduced led to an overall increase in chondrogenic capacity, evidenced by increased expression of Sox9, Col2a1, Runx2, Col10a1, and Alpl (Fig. 4*B*). Reciprocally, CM obtained from differentiating ATDC5 donor cultures in which viperin levels were overexpressed displayed an inhibitory effect on the chondrogenic differentiation capacity, evidenced by reduced Sox9, Col2a1, Runx2, Col10a1, and Alpl expression (Fig. 4*C*). The stimulatory and inhibitory actions of these CM on chondrogenic differentiation were further confirmed by determination of the glycosaminoglycan (GAG) content of these cultures. Differentiation of ATDC5 in CM obtained from donor cultures with reduced viperin levels caused an increased GAG content, whereas differentiation of ATDC5 in CM obtained from donor cultures in which viperin was overexpressed led to a reduced GAG content (Fig. 4*D*). These results together indicate that alterations in viperin levels during chondrogenic differentiation change the cell's secretome with important functional consequences for differentiation.

### Differential CXCL10 levels in viperin knockdown and overexpression secretomes

**Figure 5.**
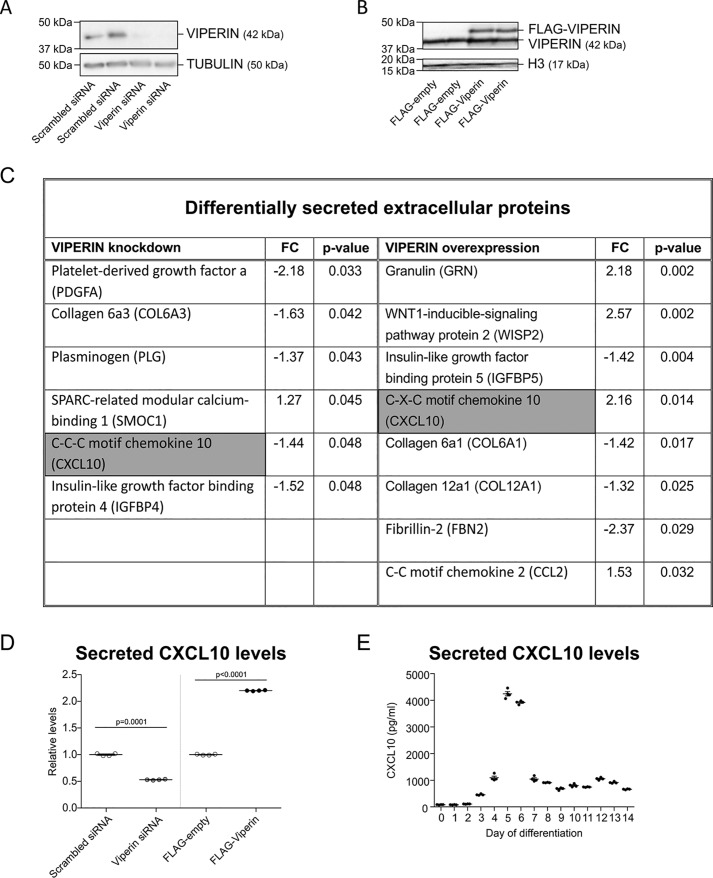
**The viperin secretome and CXCL10.** At day 5 in ATDC5 chondrogenic differentiation, viperin expression was reduced by either transfection of a viperin-specific siRNA duplex (Viperin siRNA) or a scrambled control siRNA duplex (Scrambled siRNA); or viperin expression was further induced by transfection of a p3xFLAG-viperin plasmid (FLAG-Viperin) or the empty p3xFLAG plasmid (FLAG-empty). The cells were then further differentiated until day 7, and viperin expression levels were determined (*A* and *B*). Immunoblots are shown for two independent representative examples. Culture supernatants from these day 7 cultures were collected, and the protein secretome was determined by label-free LC-MS/MS. Differentially secreted extracellular protein species (control *versus* condition) with *p* < 0.05 are shown (*C*). *FC*, fold change. In culture supernatants analyzed with LC-MS/MS, secreted CXCL10 levels were determined (*D*). In ATDC5 culture supernatants from Fig. 2, secreted CXCL10 levels were determined (*E*). The *p* values are indicated, and *error bars* indicate means ± S.E.

Because media obtained from donor cultures with reduced or overexpressed viperin levels enhance or inhibit chondrogenic differentiation (respectively), we postulated that this was caused by a differential protein composition of their secretomes. To determine the differential proteome of the conditioned culture supernatants obtained from differentiating ATDC5 cultures with reduced or overexpressed viperin levels, we undertook a label-free MS proteomics approach using LC-MS/MS. When comparing the conditioned culture supernatants of differentiating ATDC5 cultures in which a scrambled siRNA was transfected or in which viperin levels were reduced using the viperin siRNA (Fig. 5*A*), we identified six protein species that were differentially expressed in the CM (Fig. 5*C*). When comparing the conditioned culture supernatants of differentiating ATDC5 cultures in which an empty FLAG vector was transfected or in which viperin levels were overexpressed using the FLAG-viperin plasmid (Fig. 5*B*), we identified eight differentially expressed proteins in the CM (Fig. 5*C*). Interestingly, CXCL10 (*gray highlighting* in Fig. 5*C*) was the only protein species that was detected in both differential secretome proteomes. CXCL10 was decreased in CM obtained from cultures in which viperin levels were reduced and was increased in conditioned medium obtained from cultures in which viperin levels were overexpressed. Differential CXCL10 protein levels in these culture supernatants were independently confirmed by ELISA (Fig. 5*D*). Because expression of both viperin and CXCL10 are interferon-dependent (1, 17) (Fig. S1), we next verified CXCL10 secretion dynamics during chondrogenic differentiation of ATDC5. Using an ELISA, supernatant samples from cultures in Fig. 2 were analyzed for CXCL10 protein levels, and we found that CXCL10 levels in culture supernatant (Fig. 5*E*) during ATDC5 chondrogenic differentiation mirrored viperin expression dynamics (Fig. 2, *E* and *F*). The data together demonstrate that alterations in viperin levels during chondrogenic differentiation lead to specific differences in the protein composition of the chondrocyte's secretome, with CXCL10 a shared factor between the differential viperin-dependent secretomes.

### CXCL10 inhibits chondrogenic differentiation

**Figure 6.**
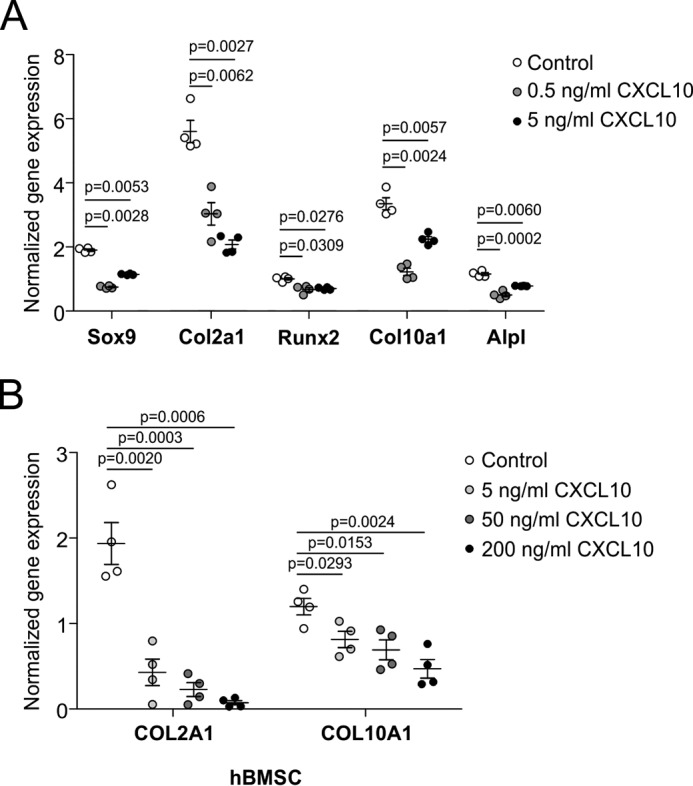
**CXCL10 attenuates chondrogenic differentiation.** ATDC5 cells were differentiated and exposed to mCXCL10 from day 5 until day 7. RNA was isolated, and expression of Sox9, Col2a1, Runx2, Col10a1, and Alpl mRNAs was determined (*A*). hBMSCs were differentiated in the chondrogenic lineage for 7 days and exposed to hCXCL10 until day 21. RNA was isolated, and expression of COL2A1 and COL10A1 mRNAs was determined (*B*). ATDC5 data were acquired from four biological replicates and normalized to β-actin mRNA levels, and individual normalized values are presented in dot plots. hBMSC gene expression data were normalized to CYCLOPHILIN mRNA levels. Individual data points in dot plots represent the average values of four biological replicates of one hBMSC donor. For statistical evaluation, an independent samples *t* test was performed relative to the corresponding controls using GraphPad Prism 5. The *p* values are indicated, and *error bars* show means ± S.E. The graphs show representative examples of three individual experiments.

Conditioned culture medium obtained from differentiating ATDC5 cultures in which viperin levels were overexpressed was found to inhibit chondrogenic differentiation. In addition to a limited number of other differentially expressed proteins, CXCL10 was the only shared protein species that was differentially present in the viperin-dependent secretomes and was increased in viperin overexpression culture supernatant. We therefore postulated that CXCL10 contributes to the inhibitory action on chondrogenic differentiation of the viperin overexpression culture supernatant (Fig. 4*C*). To determine whether CXCL10 inhibits chondrogenic differentiation, differentiating ATDC5 cultures were exposed to exogenously added mCXCL10 from day 5 of differentiation until day 7 of differentiation (the main time frame in which we observed peak expression of viperin and peak CXCL10 concentrations; Figs. 2, *E* and *F*, and 5*E*). Analysis showed that exogenously added mCXCL10 attenuated chondrogenic differentiation, evidenced by decreased expression of Sox9, Col2a1, Runx2, Col10a1, and Alpl (Fig. 6*A*). To verify the inhibitory action of CXCL10 in an independent model for chondrogenic differentiation, primary hBMSCs were differentiated into the chondrogenic lineage and exposed to increasing concentrations hCXCL10 from day 7 (peak viperin expression in hBMSC chondrogenic differentiation; Fig. 2*I*) in chondrogenic differentiation onward. CXCL10 inhibited chondrogenic differentiation of hBMSCs, evidenced by a decline of COL2A1 and COL10A1 mRNA expression (Fig. 6*B*). These data indicate that viperin-dependent alterations in secreted CXCL10 levels influence chondrogenic differentiation capacity.

### Viperin levels and CXCL10 change SMAD2/3-dependent TGF-β activity during chondrogenic differentiation of ATDC5

**Figure 7.**
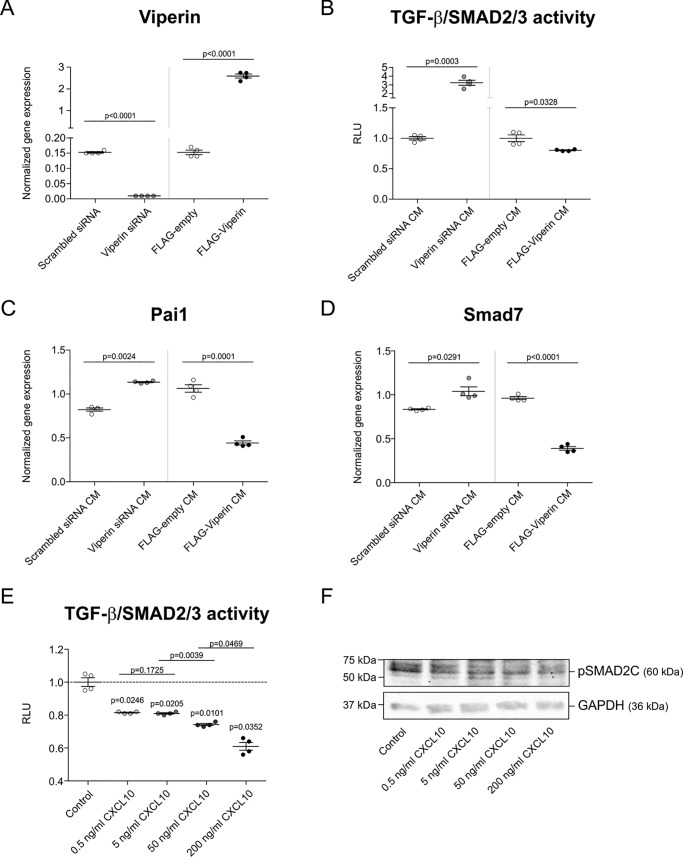
**TGF-β/SMAD2/3 activity is controlled by the viperin secretome and CXCL10.** At day 5 in ATDC5 chondrogenic differentiation, viperin expression was either reduced by transfection of a viperin-specific siRNA duplex (Viperin siRNA) or a scrambled control siRNA duplex (Scrambled siRNA) or further induced by transfection of a p3xFLAG-viperin plasmid (FLAG-Viperin) or the empty p3xFLAG plasmid (FLAG-empty). The cells were then further differentiated until day 7, and viperin expression levels were determined (*A*). Conditioned culture supernatants (CM) from these day 7 cultures were collected. Proliferating ATDC5 were used as a TGF-β/SMAD2/3 bioassay by co-transfecting a CAGA-12 firefly luciferase TGF-β/SMAD3 reporter and pGluc-CMV plasmid. The TGF-β/SMAD2/3 bioassay was then exposed to the CM supernatants for 24 h, and supernatant and cells were collected for bioluminescence analyses. Firefly bioluminescence was normalized to the Gaussia signal (*B*). Gene expression of downstream TGF-β target genes Pai1 and Smad7 was determined on samples from Fig. 4*B*/C (*C* and *D*). ATDC5 cells were differentiated and co-transfected with a CAGA-12 firefly luciferase TGF-β/SMAD3 reporter and pGluc-CMV plasmid on day 5 in chondrogenic differentiation. At day 6 in differentiation, the cells were exposed to 0.5, 5, 50, or 200 ng/ml recombinant mouse CXCL10 for 24 h until day 7. Culture supernatant and cells were collected for bioluminescence analyses. Firefly bioluminescence was normalized to the Gaussia signal (*E*). ATDC5 cells were differentiated until day 5 and then exposed to 0.5, 5, 50, or 200 ng/ml mouse CXCL10 until day 7. Protein extracts were separated by SDS-PAGE and electroblotted on membranes, followed by pSMAD2C immunodetection. GAPDH was used as a loading control (*F*). All quantitative data were acquired from four biological replicates. RT-qPCR data were normalized to β-actin mRNA levels, and individual normalized values are presented in dot plots. Bioluminescence data are presented as normalized RLUs. RLUs of controls were set at 1, and RLUs of conditions were calculated relative to the control RLUs. For statistical evaluation, an independent samples *t* test was performed relative to the corresponding controls using GraphPad Prism 5. The *p* values are indicated, and *error bars* represent means ± S.E. Graphs are representative examples of three individual experiments.

The chondro-promotive and -inhibitory actions of CM obtained from donor cultures in which viperin expression was altered, as well as the chondro-inhibitory action of CXCL10, lead to an overall inhibited or increased expression of chondrogenic as well as hypertrophic marker genes. This indicates that the activity of a basic molecular pathway responsible for chondrogenic differentiation is altered, and we therefore postulated the involvement of the major chondrogenic TGF-β/SMAD2/3 pathway. Viperin levels were either reduced or overexpressed in differentiating ATDC5 cultures from day 5 to day 7 (Fig. 7*A*), and CMs were collected. Subsequently, proliferating ATDC5 cells were used as a bioassay for SMAD2/3-dependent TGF-β activity by transfection of a CAGA12-luciferase reporter plasmid (18, 19). This TGF-β/SMAD2/3 reporter bioassay was then exposed to the above CM to determine their TGF-β/SMAD2/3 activity–modulating action. We found that the CM obtained from differentiating ATDC5 cultures in which viperin levels were reduced displayed a TGF-β/SMAD2/3 activity–promoting action (Fig. 7*B*). In contrast, CM obtained from differentiating ATDC5 cultures in which viperin levels were overexpressed displayed a TGF-β/SMAD2/3 activity–attenuating action (Fig. 7*B*). In concert with these findings, Pai1 and SMAD7 mRNA levels (TGF-β/SMAD2/3 target genes (18, 20)) were increased or decreased when ATDC5 was differentiated in CM (same as utilized in experiments shown in Fig. 4, *B* and *C*) from viperin knockdown or overexpression cultures, respectively (Fig. 7, *C* and *D*). Subsequently, we determined whether differential CXCL10 levels directly alter TGF-β/SMAD2/3 activity during chondrogenic differentiation. ATDC5 cells were transfected with the CAGA12-luciferase reporter plasmid at day 5 in chondrogenic differentiation. Then starting at day 6, the cells were exposed to increasing concentrations of recombinant murine CXCL10 and sampled for bioluminescent analyses 24 h later. The data show that CXCL10 dose-dependently reduces TGF-β/SMAD2/3 reporter activity in these cultures (Fig. 7*E*). Immunoblotting for SMAD2 phosphorylation (pSMAD2C) further showed that CXCL10 inhibits SMAD2 phosphorylation during ATDC5 chondrogenic differentiation (Fig. 7*F*), which is in concert with the observed reduction of TGF-β/SMAD2/3 reporter activity. Together these results demonstrate that alterations in viperin expression levels and CXCL10 impact SMAD2/3-dependent TGF-β activity during chondrogenic differentiation. This is in concert with the observed impact of these conditions on chondrogenic differentiation capacity (Figs. 4, *B–D*, and 6).

### Impaired chondrogenic transdifferentiation of fibroblasts from CHH patients is associated with increased viperin and CXCL10 levels

**Figure 8.**
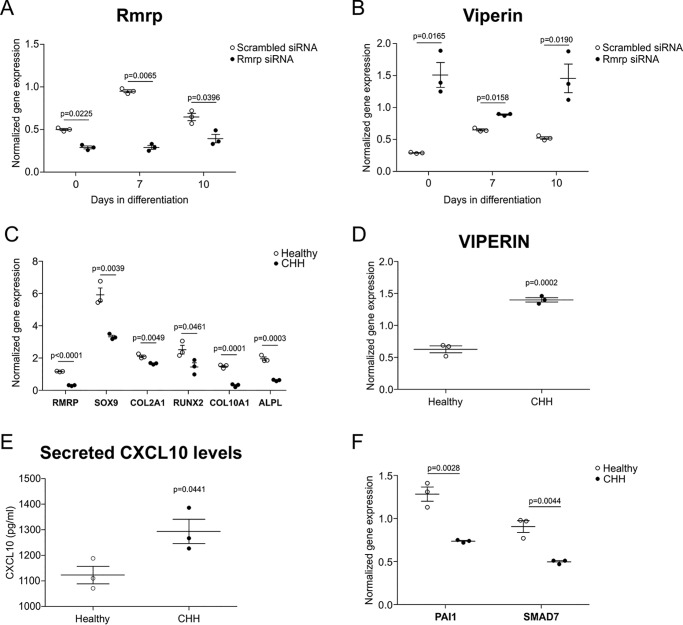
**The viperin–CXCL10–TGF-β/SMAD2/3 axis is deregulated in chondrocytic CHH cells.** Rmrp RNA expression was reduced in ATDC5 cells by transfection of a specific siRNA duplex on day −1, day 2, and day 5 during chondrogenic differentiation. A scrambled siRNA was used as control. Samples were harvested for RT-qPCR analysis at day 0, 7, or 10 in differentiation. Expression of Rmrp (*A*) and viperin (*B*) was determined at indicated time points. The data were normalized to β-actin mRNA levels, and individual normalized values are presented in dot plots. The data were acquired from three biological replicates. An independent samples *t* test was performed relative to scrambled control using GraphPad Prism 5. The *p* values are indicated, and *error bars* represent means ± S.E. Human dermal fibroblasts from three CHH patients (RMRP alleles of CHH patients carried following mutations: 127G → A and 261 C → G; 4 C → T and 77C → T; 70 A → G and 70A → G), and three healthy controls were transdifferentiated into the chondrogenic lineage by hyperconfluent plating in wells coated with Aggrecan (21, 48). RNA was isolated at day 3 of transdifferentiation, and gene expression of RMRP, SOX9, COL2A1, RUNX2, COL10A1, and ALPL mRNAs was determined (*C*). Gene expression of VIPERIN (*D*) and of PAI1 and SMAD7 (*F*) was determined in samples from *C*. Supernatants were collected from these cultures, and secreted CXCL10 protein was determined with ELISA (*E*). Gene expression data from transdifferentiated fibroblasts was normalized to CYCLOPHILIN mRNA levels, and individual normalized values are presented in dot plots. Secreted CXCL10 data are absolute concentrations (pg/ml) and presented in dot plots. For statistical evaluation, independent sample *t* tests were performed relative to healthy controls using GraphPad Prism 5. The *p* values are indicated. *Error bars* represent the means ± S.E. Graphs are representative examples of three independent experiments.

Mutations in the RMRP gene, encoding the essential RMRP snoRNA present in the RNase MRP macromolecular protein–RNA complex (4), are the cause of cartilage-hair hypoplasia-type human skeletal dysplasias (3, 5). Viperin mRNA is a substrate for endoribonucleolytic degradation by RNase MRP (2). Taking into account the here-identified chondrogenic regulatory role of a viperin–CXCL10–TGF-β/SMAD2/3 axis, we postulated that a similar mechanism is active during the impaired chondrogenic transdifferentiation that we recently discovered in CHH fibroblasts (11). In ATDC5, reduction of RMRP snoRNA levels by transfection of an RMRP-specific siRNA (Fig. 8*A*) indeed led to the induction of viperin expression (Ref. 11 and Fig. 8*B*), recapitulating the endoribonucleolytic relationship between RNase MRP and its viperin mRNA substrate. A transdifferentiation protocol was used that drives dermal fibroblasts toward a chondrocyte-like phenotype (21). As described before (11), RMRP expression and chondrocytic transdifferentiation of CHH fibroblasts was impaired, as evidenced by inhibited expression of RMRP, SOX9, COL2A1, RUNX2, COL10A1, and ALPL (Fig. 8*C*). In concert with a CHH-associated pathological defective RNase MRP endoribonucleolytic activity, we found that viperin expression in chondrogenic transdifferentiated CHH fibroblasts was increased (Fig. 8*D*). This was accompanied with increased levels of secreted CXCL10 protein in these culture supernatants (Fig. 8*E*) and decreased expression of the TGF-β target genes PAI1 and SMAD7 (Fig. 8*F*). These data suggest that defective chondrogenic transdifferentiation of CHH fibroblasts is caused by changes in TGF-β activity, induced by alterations in the viperin–CXCL10–TGF-β/SMAD2/3 axis.

## Discussion

Viperin expression was detectable early in ATDC5 and hBMSC chondrogenic differentiation and was highly induced at days 5–7 of differentiation. In the majority of embryonal growth plate, resting zone progenitor cells viperin expression was weak, representing the first days of *in vitro* chondrogenic differentiation. Interestingly, a subpopulation of resting zone cells displayed high levels of viperin expression. Chondrogenic differentiation is synchronously initiated in cell culture, although this is not expected to be synchronous in the growth plate's resting zone. We speculate that high viperin–expressing cells in the resting zone are a representation of asynchronous initiation of chondrogenic differentiation and that these cells are in a similar differentiation phase as ATDC5 and hBMSC cells during their early differentiation, when peak expression of viperin was observed. At later stages in chondrogenic differentiation, viperin expression remained increased. This was also observed in growth plates, where proliferating and prehypertrophic chondrocytes are positive for viperin expression. Viperin expression is remarkably high in terminally differentiating growth plate chondrocytes. This was not observed in the late ATDC5 and hBMSC differentiation time points tested. ATDC5 and hBMSCs are excellent *in vitro* models for chondrogenic differentiation (13). However, these models do not enter apoptosis to fully terminally differentiate, as hypertrophic chondrocytes in the growth plate do. The spatiotemporal orchestration of cell differentiation in growth plates and the lack of such spatiotemporal cues *in vitro* may lead to the absence of this differentiation phase in *in vitro* chondrogenesis models and explain the observed difference in viperin expression compared with growth plates.

Viperin was originally discovered as an interferon-inducible protein, suggesting that induction of viperin expression during chondrogenic differentiation may be driven by an intrinsic interferon-related signaling activity (further supported by data in Fig. S1). Interferon-related signaling during chondrogenic differentiation is almost unexplored. Interferon-inducible proteins p202 (22), p204 (23), and PKR (24) have been described to be important in chondrogenic differentiation. Inhibition of JAK rescues chondrogenic differentiation in osteoarthritis-like conditions (25), and IFN-γ has been demonstrated to inhibit transcription of the COL2A1 gene (26, 27) in mature chondrocytes. STAT1 expression was previously found specifically induced at day 7 in ATDC5 chondrogenic differentiation (24), supporting an intrinsically activated interferon signaling response as explanation for viperin induction at days 5–7. Different potential mechanisms to explain viperin's antiviral actions include an iron–sulfur cluster–dependent virus inhibitory action on lipid droplets (15, 28), the inhibition of farnesyl diphosphate synthase leading to important changes in plasma membrane fluidity (29), the induction of a crystalloid ER (15), and the inhibition of protein secretion from the ER (15). Viperin has been shown to recruit IRAK1 and TRAF6 to lipid droplets in pDCs, leading to nuclear translocation of IRF7 and the production of type I interferons (30). These actions are specifically activated in response to viral infection and it is therefore surprising to find that in a nonviral context viperin is active as a regulator of cellular differentiation. Previously, alterations in viperin expression have been detected during adipogenic differentiation (31) and podocyte differentiation (32). Middle zone articular cartilage chondrocytes express higher amounts of viperin as compared with superficial zone chondrocytes (33), and viperin expression has been described in osteocytes (34). It is therefore likely that the consequences of viperin expression are not limited to differentiating chondrocytes. Until now it remained unexplored whether alterations in viperin levels could influence cellular differentiation processes. Following the findings by Hinson and Cresswell (15) that viperin overexpression inhibits protein secretion from the ER in HepG2 and 293T cells, we examined whether this was also the case during chondrogenic differentiation. We found that also in chondrogenic differentiation viperin influences the rate of protein secretion, however, in the opposite direction as compared with the findings reported by the Cresswell group. We speculate that because of different cell lineages, differentiation stages and the nonviral context, important differences occur in the viperin interactome (30) that change the manner by which viperin regulates protein secretion from the ER.

We observed that total protein secretion alters in response to viperin levels, and until now no specificity has been reported for viperin-regulated protein secretion. Therefore we expect that the protein species that we found differentially secreted following manipulation of viperin levels either are relatively unstable and inhibition of cellular protein secretion would thus cause a rapid decrease in the level of this species within the secretome or are secreted in low abundance and increased protein secretion would rapidly lead to detectable differences in the level of this protein species in the secretome. In line with a viperin-mediated intrinsic interferon response during chondrogenic differentiation, the CXCL10 we detected in our LC-MS/MS analyses is an interferon-inducible chemokine (IP-10). Interestingly, platelet-derived growth factor subunit A was also found differentially expressed in the viperin overexpression secretome and platelet-derived growth factor has been described to synergistically act with IFN-γ to induce CXCL10 expression in blood-derived macrophages (17). CXCL10 has previously been described to be expressed during ATDC5 chondrogenic differentiation (35), as well as during hBMSC chondrogenic differentiation (36). Our data demonstrate that CXCL10 inhibits chondrogenic differentiation and TGF-β signaling, which is fully in line with the inhibitory action of viperin-overexpression CM on chondrogenic differentiation.

Because we utilized conditioned media as a means to study the secretomic consequences of alterations in viperin expression during chondrogenic differentiation, we cannot distinguish paracrine from autocrine secreted signals. However, it is currently not clear why chondrogenic differentiation is regulated via viperin. Control over the activity of differentiation signals in the early differentiation phase may be required to enable the cell to undertake chromatin remodeling and coordinated transcriptomic reprogramming, before it can adopt a fully differentiated phenotype. We expect that temporal para- and/or autocrine viperin-dependent CXCL10 secretion may aid in this by antagonizing TGF-β signaling and pacing cellular differentiation. Binding of CXCL10 with its CXCR3 receptor is known to activate PI3K in human airway epithelial cells (37), and recently, PI3K activity has been shown to be involved in attenuation of SMAD2/3 activity (38). Indeed we observed that CXCL10 inhibits phosphorylation of SMAD2, providing a potential mechanistic link between viperin-dependent CXCL10 secretion and TGF-β–driven chondrogenic differentiation. Alternatively, cross-talk has been identified between IFN-γ and TGF-β signaling in which IFN-γ-dependent STAT1 activity antagonizes SMAD3-dependent TGF-β signaling (39).

With a number of varying RNase MRP substrate RNAs, it would not be likely that all aspects of CHH pathobiology are caused by defective processing of one specific substrate. Instead, defective processing of one or the other specific RNase MRP substrate RNA will have different implications for different tissue/cell types. CHH-related defective processing of viperin mRNA leads to aberrant viperin levels in chondrocytic cells as shown in this study. Viperin responses (40) and CXCL10 (41) have been implicated in T-cell function, and in a microarray mRNA profiling, it was found that viperin was one of the highest differentially expressed genes up-regulated in CHH leukocytes (10), together with an enrichment of other interferon-related genes. Because CHH also presents with defective immunity caused by T-cell deficiency (42) and together with our findings that viperin and CXCL10 regulate chondrogenic differentiation, is thus tempting to speculate that interferon-related signaling through viperin and via CXCL10 is an important aspect of the molecular mechanism leading to growth plate and T-cell defects observed in CHH.

**Figure 9.**
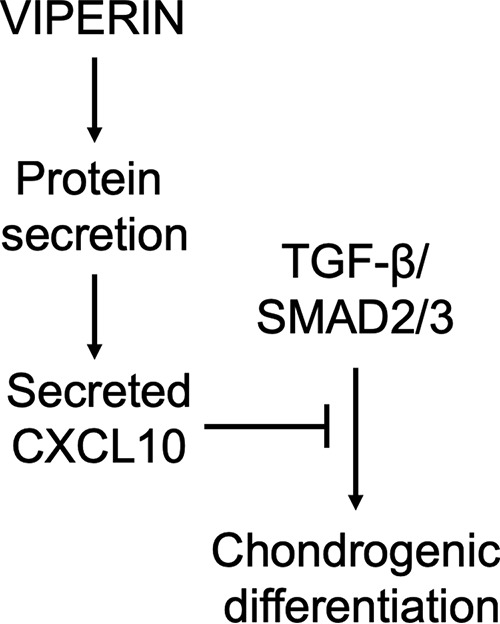
**Model of the interactions between viperin and chondrogenic differentiation.** A schematic model of the interactions between viperin and chondrogenic differentiation, suggested by our results. Viperin regulates protein secretion and controls the level of secreted CXCL10. CXCL10 inhibits TGF-β/SMAD2/3 activity, which in turn controls the level of chondrogenic differentiation.

In conclusion, our data link the antiviral protein viperin to chondrogenic development via a viperin–CXCL10–TGF-β/SMAD2/3 axis (Fig. 9), and show that a similar molecular mechanism is deregulated in CHH chondrocytic cells. For the first time we identified a molecular route that may clarify impaired chondrogenic differentiation of CHH patient cells.

## Experimental procedures

### Immunohistochemistry

5-μm-thick formalin-fixed paraffin-embedded tissue sections were prepared from embryonic day 15.5 NMRI mouse embryos. Use of the embryos was approved by the University of Freiburg (number X-14/10H), according to German law, and methods utilized to obtain the embryos were carried out in accordance with German law. Sections were deparaffinized in a xylene/ethanol series ending in PBS (136 mm NaCl (Merck Millipore), 2.7 mm KCl (Merck Millipore), 9.0 mm Na_2_PO_4_.H_2_O (Merck Millipore), 1.8 mm KH_2_PO_4_ (Merck Millipore)). For antigen retrieval, the sections were incubated in hot citrate buffer (1.8 mm citric acid (Sigma–Aldrich) and 8.2 mm trisodium citrate (VWR Prolabo, Amsterdam, The Netherlands)) for 30 min. Endogenous peroxidase activity was inactivated using peroxidase-blocking solution (Dako, Troy, MI). Slides were blocked with 10% normal sheep serum in PBS-T (0.1% Tween 20; Sigma–Aldrich). Primary antibody was incubated for 2 h at room temperature. Mouse monoclonal anti-Viperin (Merck Millipore catalog no. MABF106) was used at a 1:200 dilution. IgG2a (Dako) was used as an isotype control. Bound antibodies were detected with horseradish peroxidase (HRP)-conjugated rabbit anti-mouse secondary antibodies (Dako EnVision) for 30 min at room temperature. DAB chromogen substrate (Dako) was used for detection. Sections were counterstained with hematoxylin (Dako), dehydrated, and mounted with histomount (Thermo Shandon, Waltham, MA). Microphotographs were acquired using a Zeiss Axioscope A1.

### Cell culture and differentiation of ATDC5

ATDC5 cells (12) (RIKEN BRC, Japan) were cultured in a humidified atmosphere at 37 °C, 5% CO_2_ in proliferation medium (DMEM/F-12 (Invitrogen), 5% fetal calf serum (FCS) (Sigma–Aldrich), 1% antibiotic/antimycotic (Invitrogen), and 1% nonessential amino acids (NEAA) (Invitrogen)). The cells were tested negative for potential mycoplasma infection. To induce chondrogenic differentiation, the cells were plated at 6,400 cells/cm^2^. After 24 h, chondrogenic differentiation was initiated by changing the medium to differentiation medium (proliferation medium supplemented with 10 μg/ml insulin (Sigma–Aldrich), 10 μg/ml transferrin (Roche), and 30 nm sodium selenite (Sigma–Aldrich)). Differentiation medium was refreshed every 2 days for the first 10 days and each day after day 10. In addition, ATDC5 cells were differentiated for 5 days and then exposed to 0.5 or 5 ng/ml recombinant murine CXCL10 (Peprotech, Rocky Hill, NJ, catalog no. 250-16) until day 7 in differentiation. When ATDC5 cells were differentiated using CM, the CM was refreshed every other day.

### Cell culture and differentiation of hBMSCs

hBMSCs were obtained from residual iliac crest bone marrow aspirate from young, genetically healthy, individuals undergoing spinal surgery. Approval from the Maastricht University Medical Center Medical Ethical Committee for the use of this material was obtained and assigned approval code MEC 08-4-056. Human BMSCs from four individual donors were isolated from the aspirate using Ficoll Paque (Amersham Biosciences). Proliferation medium consisted of DMEM high-glucose (Invitrogen), 10% FCS (ES grade), 1% antibiotic/antimycotic, and 1% NEAA. Passage 3 cells were plated at 30,000 cells/cm^2^ in quadruplicates, and chondrogenic differentiation was initiated the next day by changing to differentiation medium (proliferation medium supplemented with 1% insulin/transferrin/selenium–sodium pyruvate (Invitrogen), 50 μg/ml l-ascorbic acid-2-phosphate (Sigma), and 1 ng/ml TGF-β3 (R&D)) (14). Medium was changed every 2 days. Human CXCL10 (Peprotech) was used at the indicated concentrations (0.5, 5, 50, and 200 ng/ml). Cells were harvested in TRIzol at day 0, 2, 7, 14, and 21 in differentiation for RNA isolation.

### Real-time quantitative PCR (RT-qPCR)

RNA was extracted from cells with TRIzol reagent (Invitrogen) and isolated by collecting the aqueous phase after phase separation. RNA was precipitated with isopropanol (30 min, −80 °C) and centrifuged for 30 min at 15,000 rpm, 4 °C. RNA pellets were washed with 80% ethanol, and potential DNA contamination was removed by DNase I (Roche) treatment (1 h, 37 °C). After subsequent ethanol precipitation, RNA was dissolved in 15 μl of DNase/RNase-free water (Eurogentec, Seraing, Belgium). RNA quantity and purity were determined spectrophotometrically (Biodrop, Isogen Life Sciences, Utrecht, The Netherlands). Reverse transcription of RNA and RT-qPCR were performed as described previously (11). Housekeeping genes were β-actin for ATDC5 and CYCLOPHILIN for hBMSCs and human fibroblasts. Primer sequences are provided in Table 1.

**Table 1:** **RT-qPCR primer sequences** Primer sequences were designed for *Mus musculus* (*Mm*) or *Homo sapiens* (*Hs*).

Gene	Forward	Reverse
ALPL (*Hs*)	CCGTGGCAACTCTATCTTTGG	CAGGCCCATTGCCATACAG
Alpl (*Mm*)	CCGATGGCACACCTGCTT	GGAGGCATACGCCATCACAT
β-Actin (*Mm*)	CCGAGCGCGAGATCGT	TGGCCATCTCGTTCTCGAA
COL2A1 (*Hs*)	TGGGTGTTCTATTTATTTATTGTCTTCCT	GCGTTGGACTCACACCAGTTAGT
Col2a1 (*Mm*)	TGGGTGTTCTATTTATTTATTGTCTTCCT	GCGTTGGACTCACACCAGTTAGT
COL10A1 (*Hs*)	ATGATGAATACACCAAAGGCTACCT	ACGCACACCTGGTCATTTTCTG
Col10a1 (*Mm*)	CATGCCTGATGGCTTCATAAA	AAGCAGACACGGGCATACCT
CYCLOPHILIN (*Hs*)	TTCCTGCTTTCACAGAATTATTCC	GCCACCAGTGCCATTATGG
PAI1 (*Hs*)	GTCTGCTGTGCACCATCCCCCATC	TTGTCATCAATCTTGAATCCCATA
Pai1 (*Mm*)	ACGTCGTGGAACTGCCCTAC	CAGCGATGAACATGCTGAGG
RMRP (*Hs*)	GAGAGTGCCACGTGCATACG	ACGCTTCTTGGCGGACTTT
Rmrp (*Mm*)	ATACGAGGGACATGTTCCTTATCC	TTGGCGGGCTAACAGTGACT
RUNX2 (*Hs*)	TGATGACACTGCCACCTCTG	GCACCTGCCTGGCTCTTCT
Runx2 (*Mm*)	GACGAGGCAAGAGTTTCACC	GGACCGTCCACTGTCACTTT
SMAD7 (*Hs*)	CCTTAGCCGACTCTGCGAACTA	CCAGATAATTCGTTCCCCCTGT
Smad7 (*Mm*)	GCAACCCCCATCACCTTAGTC	GTTTGAGAAAATCCATTGGGTATCTG
SOX9 (*Hs*)	AGTACCCGCACCTGCACAAC	CGCTTCTCGCTCTCGTTCAG
Sox9 (*Mm*)	AGTACCCGCACCTGCACAAC	TACTTGTAGTCCGGGTGGTCTTTC
VIPERIN (*Hs*)	CCAACCAGCGTCAACTATCACTT	GGAAACAGAAGCCGCATTTG
Viperin (*Mm*)	TGCTATCTCCTGCGACAGCTT	CCTTGACCACGGCCAATC

### Immunoblotting

The cells were washed three times with 0.9% NaCl. The cells were lysed in radioimmune precipitation assay buffer (150 mm NaCl, 1% Nonidet P-40, 0.5% sodium deoxycholate, 0.1% SDS, 50 mm Tris pH 8.0, 5.0 mm EDTA, pH 8.0, 0.5 mm DTT, 1 mm phenylmethylsulfonyl fluoride, and phosphatase inhibitor (PhosSTOP; Roche)). Samples were homogenized by sonication (Soniprep 150 MSE) using the following protocol: 14 cycles of 1-s sonication followed by a 1-s interval, amplitude 10. Cell debris was removed by centrifugation. Total protein concentration was determined with a BCA assay (Sigma–Aldrich). Samples were separated by gel electrophoresis and transferred to nitrocellulose membranes by electroblotting. The following primary antibodies were used for immunodetection: mouse monoclonal anti-Viperin (Merck Millipore catalog no. MABF106; 1:500 dilution) and rabbit monoclonal anti-pSMAD2C (Cell Signaling Technologies catalog no. 3108; 1:1000 dilution). As controls, mouse monoclonal anti-Histone H3 (Abcam catalog no. 24834; 1:1000 dilution), mouse monoclonal anti–α-Tubulin (Sigma–Aldrich catalog no. T6074; 1:10,000 dilution), and mouse monoclonal anti-GAPDH (Fitzgerald catalog no. 10R-G109b; 1:5000 dilution) were used. HRP-conjugated polyclonal rabbit anti-mouse (Dako) or HRP-conjugated swine anti-rabbit (Dako) was applied as a secondary antibody, and the bound antibodies were detected by ECL.

### Transfection of siRNAs

Chondrogenic differentiating ATDC5 cells were transfected (Pepmute; SignaGen Laboratories, Rockville, MD; according to the manufacturer's protocol) with 100 nm siRNA duplexes (custom synthesized by Eurogentec; Table 2) targeting Viperin (on day 3 or 5 of chondrogenic differentiation) or Rmrp RNA (on day −1, 2, and 5 of chondrogenic differentiation). Scrambled siRNA duplex was purchased from Eurogentec; REF: SR-CL000-005.

**Table 2:** **siRNA sequences**

siRNA	Sense	Antisense
Viperin siRNA	GGGAAGCAGAAAGAUUUCUdTdT	AGAAAUCUUUCUGCUUCCCdTdT
Rmrp siRNA	CAUGUUCCUUAUCCUUUCGdTdT	CGAAAGGAUAAGGAACAUGdTdT

### Plasmids and transfection

The viperin coding sequence, flanked by NotI–XbaI restriction sites, was custom-made synthesized by GeneCust (Ellange, Luxembourg) and originally cloned in pUC57 vector and sequence verified. The insert was then subcloned into the p3xFLAG plasmid (Promega, Southampton, UK) using the NotI–XbaI restriction sites, yielding the viperin overexpression plasmid (FLAG-viperin). On day 4, 5, or 6 of ATDC5 chondrogenic differentiation, viperin expression was further induced in 6-well plates by transfection of 625 ng of FLAG-viperin plasmid or an empty plasmid (FLAG-empty) as a control, using polyethylenimine (PEI; Polysciences, Warrington, PA) transfection reagent. DNA and PEI were complexed for 10 min at room temperature in DMEM/F-12 (1.88 μl of PEI (1 μg/μl) per 625 ng construct per well) and added to the ATDC5 cells. To determine protein secretion 250 ng of CMV promoter-driven secreted Gaussia luciferase plasmid (pGluc-CMV(43)) was co-transfected with 200 ng of CMV promoter-driven firefly luciferase pGL4.20-CMV (control; Promega) on day 3 or 5 of ATDC5 chondrogenic differentiation (6-well format), using PEI transfection reagent. Culture supernatant and cells were collected for bioluminescence analyses, and Gaussia bioluminescence was normalized to the firefly signal. Viperin levels were either reduced or overexpressed in differentiating ATDC5 cultures from day 5 to day 7, and conditioned culture supernatants were collected. Subsequently, proliferating ATDC5 cells were used as a bioassay for SMAD3-dependent TGF-β activity by transfection with 500 ng of CAGA12-luciferase reporter plasmid (18) (kind gift of Dr. Dalmay (19)) and 125 ng of pGluc-CMV (43) plasmid as a transfection control, using PEI transfection reagent. The proliferating ATDC5 cells were then exposed to the above mentioned conditioned culture supernatants for 24 h to determine the TGF-β/SMAD3 activity, where firefly bioluminescence was normalized to the Gaussia signal. The action of exogenously added CXCL10 on SMAD3-dependent TGF-β activity during chondrogenic differentiation was determined by transfecting day 5 differentiating ATDC5 cells in 6-well format with 500 ng of CAGA12-luciferase reporter and 125 ng of pGluc-CMV plasmid as a transfection control, using PEI transfection reagent. Twenty-four hours later, cells were exposed to 0.5, 5, 20, or 200 ng/ml recombinant murine CXCL10 (Peprotech catalog no. 250-16). At day 7 in differentiation, culture supernatant and cells were then collected for bioluminescence analyses, and firefly bioluminescence was normalized to the Gaussia signal. pGluc-CMV (Gaussia luciferase), pGL4.20-CMV (firefly luciferase), and CAGA-12 (firefly luciferase) samples were all harvested for bioluminescence detection using the Dual Luciferase reporter assay system (Promega) as described by the manufacturer and measured on a Fluostar Omega plate reader (BMG Labtech, Ortenberg, Germany).

### Alcian Blue and Crystal Violet staining and quantification

The cells were washed twice with 0.9% NaCl and fixated with 4% paraformaldehyde in PBS for 10 min at room temperature. Fixated cells were washed six times with distilled water and air-dried. Dried fixated cells were incubated for 30 min at room temperature with respectively 1% (m/v) Alcian Blue (Acros Organics, Geel, Belgium) in 0.1 m HCl or 0.1% (m/v) Crystal Violet (Sigma–Aldrich). The cells were washed six times with distilled water and allowed to air dry. Alcian Blue was extracted by incubation with 6 m guanidine-HCl (Sigma–Aldrich) for 2 h on a plate shaker (IKA HS 260 Basic, IKA, Staufen, Germany). Crystal Violet was extracted by incubation with 10% acetic acid (VWR) for 15 min on a plate shaker (IKA). Extracted Alcian Blue and Crystal Violet were quantified spectrophotometrically at 645 and 590 nm, respectively, using a plate reader (ThermoScientific Multiskan FC, Waltham, MA). Crystal Violet (DNA content) was used as normalization for Alcian Blue GAG content.

### In-solution tryptic digestion and MS proteomics of media following viperin knockdown or overexpression

At day 5 in ATDC5 chondrogenic differentiation, viperin expression was either reduced by transfection of a viperin-specific siRNA duplex or a scrambled control siRNA duplex or further induced by transfection of the FLAG-viperin plasmid or the empty FLAG plasmid. The cells were then further differentiated until day 7, and CM was collected. During differentiation phenol red–free DMEM/F-12 (Invitrogen) was used to accommodate MS analysis. To obtain sufficient amounts of CM, the experiment was simultaneously performed in triplicate for each condition. Triplicates were then pooled to obtain three times scrambled siRNA duplex, three times viperin siRNA duplex, three times empty FLAG, and three times FLAG-viperin overexpression CM samples. Two representative triplicates from each condition were utilized to confirm viperin knockdown and overexpression by means of immunoblotting. The CM was centrifuged for 5 min at 1200 rpm to remove dead cells and subsequently for 5 min at 13,000 rpm to remove remaining debris. Then Complete Ultra protease inhibitors (EDTA-free; Roche) were added. The samples were stored at −80 °C prior to downstream analysis. Protein concentrations of CM were calculated using the Bradford assay with Coomassie Plus^TM^ protein assay reagent (Thermo Scientific, Rockford, IL), read at 660 nm. In solution tryptic digestion was undertaken as previously described (44). LC-MS/MS analysis was performed on trypsin digests using an Ultimate 3000 Nano system (Dionex, ThermoFisher Scientific, Waltham, MA) on line to a Q-Exactive Quadrupole-Orbitrap instrument (Thermo Scientific) (45). Proteins were identified using Peaks® 7 PTM software (version 7; Bioinformatics Solutions, Waterloo, Canada), searching against the UniMouse database, with a false discovery rate of 1%, a minimum of two unique peptides per protein and a confidence score of >50.

### Label-free quantification of MS proteomics data

Progenesis QI^TM^ software (version 4, Waters, Manchester, UK) was used to identify fold changes in protein abundance between scrambled siRNA duplex and viperin knockdown siRNA duplex and between the FLAG-empty and FLAG-viperin overexpression constructs as described previously (46). Only unique peptides were used for quantification and, with *p* values <0.05, were considered to be differentially expressed.

### CXCL10 ELISA

To determine CXCL10 protein concentrations in media, the mouse CXCL10/IP-10/CRG-2 DuoSet ELISA (R&D catalog no. DY466) and the human CXCL10/IP-10 Duoset ELISA (R&D catalog no. DY266) were utilized according to the manufacturer's protocol. Plates were colorimetrically measured on a ThermoScientific Multiskan FC plate reader (Waltham, MA).

### Chondrogenic transdifferentiation of human dermal fibroblasts

Human dermal fibroblasts (passage 7–9) from three CHH patients (RMRP alleles of CHH patients carried the following mutations: 127G → A and 261 C → G; 4 C → T and 77C → T; 70 A → G and 70A → G) and three healthy controls were hyperconfluently plated (100,000 cells/cm^2^) in aggrecan-coated (Sigma–Aldrich; 2.5 μg/cm^2^) wells as previously described (21). The cells were directly plated in transdifferentiation medium which consisted of DMEM/F-12 + GlutaMAX (Invitrogen), 10% FCS (PAA), 1% antibiotic/antimycotic (Invitrogen), 1% NEAA (Invitrogen) + 1% insulin/transferrin/selenium–sodium pyruvate (Life Technologies), 50 μg/ml ascorbic acid 2-phosphate (Sigma–Aldrich), and 1 ng/ml human recombinant TGF-β3 (Life Technologies). Transdifferentiation medium was refreshed on day 2, and transdifferentiated cartilaginous nodules were harvested in TRIzol on day 3 of transdifferentiation for RNA isolation. Ethical permission was obtained from the medical ethical Institutional Review Board of Freiburg University Hospital, and methods and experimental protocols to obtain dermal fibroblasts were carried out in accordance with the Freiburg University Hospital medical ethical Institutional Review Board, according to German law and abiding the Declaration of Helsinki principles.

### Statistics

Gene expression, ELISA, colorimetric, and bioluminescence data were statistically analyzed using GraphPad Prism 5 (La Jolla, CA). A two-tailed independent samples *t* test was performed relative to the corresponding control condition. Individual values are presented in graphs in dot plots, and the *p* values and means ± S.E. are indicated in the figures. The number of biological replicates and the number of repeated experiments are indicated in the figure legends. Statistical significance was defined as *p* < 0.05.

### Data availability

The MS proteomics data have been deposited to the ProteomeXchange Consortium via the PRIDE partner repository (47) with data set identifier PXD006999. All other relevant data are available from the authors.

## Author contributions

M. M. F. S., M. M. J. C., B. Z., L. W. v. R., M. J. P., and T. J. M. W. conceptualization; M. M. F. S., M. M. J. C., D. A. M. S., G. G. H. v. d. A., P. J. v. D., F. F., M. J. P., and T. J. M. W. data curation; M. M. F. S., M. M. J. C., M. J. P., and T. J. M. W. software; M. M. F. S., M. M. J. C., D. A. M. S., G. G. H. v. d. A., M. J. P., and T. J. M. W. formal analysis; M. M. F. S., M. M. J. C., D. A. M. S., G. G. H. v. d. A., M. J. P., and T. J. M. W. validation; M. M. F. S., M. M. J. C., D. A. M. S., G. G. H. v. d. A., P. J. v. D., F. F., and M. J. P. investigation; M. M. F. S., M. M. J. C., G. G. H. v. d. A., and T. J. M. W. visualization; M. M. F. S., M. M. J. C., D. A. M. S., G. G. H. v. d. A., P. J. v. D., F. F., B. Z., M. J. P., and T. J. M. W. methodology; M. M. F. S., M. M. J. C., D. A. M. S., P. J. v. D., F. F., B. Z., L. W. v. R., M. J. P., and T. J. M. W. writing-original draft; M. M. F. S., M. M. J. C., G. G. H. v. d. A., M. J. P., and T. J. M. W. writing-review and editing; B. Z., L. W. v. R., M. J. P., and T. J. M. W. resources; B. Z., L. W. v. R., M. J. P., and T. J. M. W. supervision; B. Z., L. W. v. R., M. J. P., and T. J. M. W. funding acquisition; B. Z., L. W. v. R., M. J. P., and T. J. M. W. project administration.

## Supplementary Material

Supporting Information

## Footnotes

3 The abbreviations used are:

ER: endoplasmic reticulum
MRP: mitochondrial RNA processing
RT-qPCR: real-time quantitative PCR
snoRNA: small nucleolar RNA
CHH: cartilage-hair hypoplasia
TGF: transforming growth factor
hBMSC: human bone marrow stem cell
CM: conditioned medium
GAG: Glycosaminoglycan
IFN: interferon
HRP: horseradish peroxidase
DMEM: Dulbecco's modified Eagle's medium
FCS: fetal calf serum
NEAA: nonessential amino acid(s)
PEI: polyethylenimine
CMV: cytomegalovirus
RLU: relative light unit.

## References

[1] Seo J. Y., Yaneva R., and Cresswell P. (2011) Viperin: a multifunctional, interferon-inducible protein that regulates virus replication. Cell Host Microbe 10, 534–539 10.1016/j.chom.2011.11.004 22177558 PMC3246677

[2] Mattijssen S., Hinson E. R., Onnekink C., Hermanns P., Zabel B., Cresswell P., and Pruijn G. J. (2011) Viperin mRNA is a novel target for the human RNase MRP/RNase P endoribonuclease. Cell Mol. Life Sci 68, 2469–2480 10.1007/s00018-010-0568-3 21053045 PMC3121944

[3] Ridanpää M., van Eenennaam H., Pelin K., Chadwick R., Johnson C., Yuan B., vanVenrooij W., Pruijn G., Salmela R., Rockas S., Mäkitie O., Kaitila I., and de la Chapelle A. (2001) Mutations in the RNA component of RNase MRP cause a pleiotropic human disease, cartilage-hair hypoplasia. Cell 104, 195–203 10.1016/S0092-8674(01)00205-7 11207361

[4] Mattijssen S., Welting T. J., and Pruijn G. J. (2010) RNase MRP and disease. Wiley Interdiscip. Rev. RNA 1, 102–116 10.1002/wrna.9 21956908

[5] Makitie O., and Kostjukovits S. (1993) Cartilage-hair hypoplasia: anauxetic dysplasia spectrum disorders. In GeneReviews(R) (Pagon R. A., Adam M. P., Ardinger H. H., Wallace S. E., Amemiya A., Bean L. J. H., Bird T. D., Ledbetter N., Mefford H. C., Smith R. J. H., and Stephens K., eds) Seattle, WA 22420014

[6] Baron J., Sävendahl L., De Luca F., Dauber A., Phillip M., Wit J. M., and Nilsson O. (2015) Short and tall stature: a new paradigm emerges. Nat Rev Endocrinol 11, 735–746 10.1038/nrendo.2015.165 26437621 PMC5002943

[7] Gill T., Cai T., Aulds J., Wierzbicki S., and Schmitt M. E. (2004) RNase MRP cleaves the CLB2 mRNA to promote cell cycle progression: novel method of mRNA degradation. Mol. Cell. Biol. 24, 945–953 10.1128/MCB.24.3.945-953.2004 14729943 PMC321458

[8] Chang D. D., and Clayton D. A. (1987) A mammalian mitochondrial RNA processing activity contains nucleus-encoded RNA. Science 235, 1178–1184 10.1126/science.2434997 2434997

[9] Goldfarb K. C., and Cech T. R. (2017) Targeted CRISPR disruption reveals a role for RNase MRP RNA in human preribosomal RNA processing. Genes Dev. 31, 59–71 10.1101/gad.286963.116 28115465 PMC5287113

[10] Hermanns P., Bertuch A. A., Bertin T. K., Dawson B., Schmitt M. E., Shaw C., Zabel B., and Lee B. (2005) Consequences of mutations in the non-coding RMRP RNA in cartilage-hair hypoplasia. Hum. Mol. Genet. 14, 3723–3740 10.1093/hmg/ddi403 16254002

[11] Steinbusch M. M. F., Caron M. M. J., Surtel D. A. M., Friedrich F., Lausch E., Pruijn G. J. M., Verhesen W., Schroen B. L. M., van Rhijn L. W., Zabel B., and Welting T. J. M. (2017) Expression of RMRP RNA is regulated in chondrocyte hypertrophy and determines chondrogenic differentiation. Sci. Rep. 7, 6440 10.1038/s41598-017-06809-5 28743979 PMC5527100

[12] Atsumi T., Miwa Y., Kimata K., and Ikawa Y. (1990) A chondrogenic cell line derived from a differentiating culture of AT805 teratocarcinoma cells. Cell Differ. Dev 30, 109–116 10.1016/0922-3371(90)90079-C 2201423

[13] Yao Y., and Wang Y. (2013) ATDC5: an excellent *in vitro* model cell line for skeletal development. J. Cell. Biochem. 114, 1223–1229 10.1002/jcb.24467 23192741

[14] Caron M. M., Emans P. J., Surtel D. A., Cremers A., Voncken J. W., Welting T. J., and van Rhijn L. W. (2012) Activation of NF-κB/p65 facilitates early chondrogenic differentiation during endochondral ossification. PLoS One 7, e33467 10.1371/journal.pone.0033467 22428055 PMC3299787

[15] Hinson E. R., and Cresswell P. (2009) The N-terminal amphipathic α-helix of viperin mediates localization to the cytosolic face of the endoplasmic reticulum and inhibits protein secretion. J. Biol. Chem. 284, 4705–4712 10.1074/jbc.M807261200 19074433 PMC2640954

[16] Kronenberg H. M. (2003) Developmental regulation of the growth plate. Nature 423, 332–336 10.1038/nature01657 12748651

[17] Dhillon N. K., Peng F., Ransohoff R. M., and Buch S. (2007) PDGF synergistically enhances IFN-γ–induced expression of CXCL10 in blood-derived macrophages: implications for HIV dementia. J. Immunol. 179, 2722–2730 10.4049/jimmunol.179.5.2722 17709485

[18] Dennler S., Itoh S., Vivien D., ten Dijke P., Huet S., and Gauthier J. M. (1998) Direct binding of Smad3 and Smad4 to critical TGF β-inducible elements in the promoter of human plasminogen activator inhibitor-type 1 gene. EMBO J. 17, 3091–3100 10.1093/emboj/17.11.3091 9606191 PMC1170648

[19] Pais H., Nicolas F. E., Soond S. M., Swingler T. E., Clark I. M., Chantry A., Moulton V., and Dalmay T. (2010) Analyzing mRNA expression identifies Smad3 as a microRNA-140 target regulated only at protein level. RNA 16, 489–494 10.1261/rna.1701210 20071455 PMC2822914

[20] Nakao A., Afrakhte M., Morén A., Nakayama T., Christian J. L., Heuchel R., Itoh S., Kawabata M., Heldin N. E., Heldin C. H., and ten Dijke P. (1997) Identification of Smad7, a TGFβ-inducible antagonist of TGF-β signalling. Nature 389, 631–635 10.1038/39369 9335507

[21] French M. M., Rose S., Canseco J., and Athanasiou K. A. (2004) Chondrogenic differentiation of adult dermal fibroblasts. Ann. Biomed. Eng. 32, 50–56 10.1023/B:ABME.0000007790.65773.e0 14964721

[22] Kong L., and Liu C. J. (2008) Mediation of chondrogenic and osteogenic differentiation by an interferon-inducible p202 protein. Cell Mol. Life Sci. 65, 3494–3506 10.1007/s00018-008-8342-5 18791844 PMC11131663

[23] Zhang Y., Kong L., Carlson C. S., and Liu C. J. (2008) Cbfa1-dependent expression of an interferon-inducible p204 protein is required for chondrocyte differentiation. Cell Death Differ. 15, 1760–1771 10.1038/cdd.2008.112 18636074

[24] Morimoto H., Baba R., Haneji T., and Doi Y. (2013) Double-stranded RNA-dependent protein kinase regulates insulin-stimulated chondrogenesis in mouse clonal chondrogenic cells, ATDC-5. Cell Tissue Res. 351, 41–47 10.1007/s00441-012-1521-6 23180319

[25] van Beuningen H. M., de Vries-van Melle M. L., Vitters E. L., Schreurs W., van den Berg W. B., van Osch G. J., and van der Kraan P. M. (2014) Inhibition of TAK1 and/or JAK can rescue impaired chondrogenic differentiation of human mesenchymal stem cells in osteoarthritis-like conditions. Tissue Eng. Part A 20, 2243–2252 10.1089/ten.tea.2013.0553 24547725 PMC4137338

[26] Osaki M., Tan L., Choy B. K., Yoshida Y., Cheah K. S., Auron P. E., and Goldring M. B. (2003) The TATA-containing core promoter of the type II collagen gene (COL2A1) is the target of interferon-γ–mediated inhibition in human chondrocytes: requirement for Stat1α, Jak1 and Jak2. Biochem. J. 369, 103–115 10.1042/bj20020928 12223098 PMC1223055

[27] Goldring M. B., Fukuo K., Birkhead J. R., Dudek E., and Sandell L. J. (1994) Transcriptional suppression by interleukin-1 and interferon-γ of type II collagen gene expression in human chondrocytes. J. Cell. Biochem. 54, 85–99 10.1002/jcb.240540110 8126089

[28] Hinson E. R., and Cresswell P. (2009) The antiviral protein, viperin, localizes to lipid droplets via its N-terminal amphipathic α-helix. Proc. Natl. Acad. Sci. U.S.A. 106, 20452–20457 10.1073/pnas.0911679106 19920176 PMC2778571

[29] Wang X., Hinson E. R., and Cresswell P. (2007) The interferon-inducible protein viperin inhibits influenza virus release by perturbing lipid rafts. Cell Host Microbe 2, 96–105 10.1016/j.chom.2007.06.009 18005724

[30] Saitoh T., Satoh T., Yamamoto N., Uematsu S., Takeuchi O., Kawai T., and Akira S. (2011) Antiviral protein Viperin promotes Toll-like receptor 7- and Toll-like receptor 9-mediated type I interferon production in plasmacytoid dendritic cells. Immunity 34, 352–363 10.1016/j.immuni.2011.03.010 21435586

[31] Satish L., Krill-Burger J. M., Gallo P. H., Etages S. D., Liu F., Philips B. J., Ravuri S., Marra K. G., LaFramboise W. A., Kathju S., and Rubin J. P. (2015) Expression analysis of human adipose-derived stem cells during in vitro differentiation to an adipocyte lineage. BMC Med. Genomics 8, 41 10.1186/s12920-015-0119-8 26205789 PMC4513754

[32] Li Z., Yin H., Hao S., Wang L., Gao J., Tan X., and Yang Z. (2016) miR-200 family promotes podocyte differentiation through repression of RSAD2. Sci. Rep. 6, 27105 10.1038/srep27105 27251424 PMC4890021

[33] Amanatullah D. F., Yamane S., and Reddi A. H. (2014) Distinct patterns of gene expression in the superficial, middle and deep zones of bovine articular cartilage. J. Tissue Eng. Regen. Med. 8, 505–514 22777751 10.1002/term.1543

[34] Grewal T. S., Genever P. G., Brabbs A. C., Birch M., and Skerry T. M. (2000) Best5: a novel interferon-inducible gene expressed during bone formation. FASEB J. 14, 523–531 10.1096/fasebj.14.3.523 10698968

[35] Osawa A., Kato M., Matsumoto E., Iwase K., Sugimoto T., Matsui T., Ishikura H., Sugano S., Kurosawa H., Takiguchi M., and Seki N. (2006) Activation of genes for growth factor and cytokine pathways late in chondrogenic differentiation of ATDC5 cells. Genomics 88, 52–64 10.1016/j.ygeno.2006.02.013 16597497

[36] Cristino S., Piacentini A., Manferdini C., Codeluppi K., Grassi F., Facchini A., and Lisignoli G. (2008) Expression of CXC chemokines and their receptors is modulated during chondrogenic differentiation of human mesenchymal stem cells grown in three-dimensional scaffold: evidence in native cartilage. Tissue Eng. Part A 14, 97–105 10.1089/ten.a.2007.0121,10.1089/ten.tec.2007.0300,10.1089/ten.2007.0121 18333808

[37] Shahabuddin S., Ji R., Wang P., Brailoiu E., Dun N., Yang Y., Aksoy M. O., and Kelsen S. G. (2006) CXCR3 chemokine receptor-induced chemotaxis in human airway epithelial cells: role of p38 MAPK and PI3K signaling pathways. Am. J. Physiol. Cell Physiol. 291, C34–C39 10.1152/ajpcell.00441.2005 16467404

[38] Yu J. S., Ramasamy T. S., Murphy N., Holt M. K., Czapiewski R., Wei S. K., and Cui W. (2015) PI3K/mTORC2 regulates TGF-β/activin signalling by modulating Smad2/3 activity via linker phosphorylation. Nat. Commun. 6, 7212 10.1038/ncomms8212 25998442 PMC4455068

[39] Ulloa L., Doody J., and Massagué J. (1999) Inhibition of transforming growth factor-β/SMAD signalling by the interferon-γ/STAT pathway. Nature 397, 710–713 10.1038/17826 10067896

[40] Qiu L. Q., Cresswell P., and Chin K. C. (2009) Viperin is required for optimal Th2 responses and T-cell receptor-mediated activation of NF-κB and AP-1. Blood 113, 3520–3529 10.1182/blood-2008-07-171942 19047684

[41] Groom J. R., and Luster A. D. (2011) CXCR3 in T cell function. Exp. Cell Res. 317, 620–631 10.1016/j.yexcr.2010.12.017 21376175 PMC3065205

[42] Mäkitie O., Kaitila I., and Savilahti E. (1998) Susceptibility to infections and *in vitro* immune functions in cartilage-hair hypoplasia. Eur. J. Pediatr. 157, 816–820 10.1007/s004310050943 9809821

[43] Groot A. J., Habets R., Yahyanejad S., Hodin C. M., Reiss K., Saftig P., Theys J., and Vooijs M. (2014) Regulated proteolysis of NOTCH2 and NOTCH3 receptors by ADAM10 and presenilins. Mol. Cell. Biol. 34, 2822–2832 10.1128/MCB.00206-14 24842903 PMC4135574

[44] Peffers M. J., Beynon R. J., and Clegg P. D. (2013) Absolute quantification of selected proteins in the human osteoarthritic secretome. Int. J. Mol. Sci. 14, 20658–20681 10.3390/ijms141020658 24132152 PMC3821636

[45] Thorpe C. T., Peffers M. J., Simpson D., Halliwell E., Screen H. R., and Clegg P. D. (2016) Anatomical heterogeneity of tendon: Fascicular and interfascicular tendon compartments have distinct proteomic composition. Sci. Rep. 6, 20455 10.1038/srep20455 26842662 PMC4740843

[46] Peffers M. J., Thorpe C. T., Collins J. A., Eong R., Wei T. K., Screen H. R., and Clegg P. D. (2014) Proteomic analysis reveals age-related changes in tendon matrix composition, with age- and injury-specific matrix fragmentation. J. Biol. Chem. 289, 25867–25878 10.1074/jbc.M114.566554 25077967 PMC4162187

[47] Vizcaíno J. A., Côté R. G., Csordas A., Dianes J. A., Fabregat A., Foster J. M., Griss J., Alpi E., Birim M., Contell J., O'Kelly G., Schoenegger A., Ovelleiro D., Pérez-Riverol Y., Reisinger F., et al (2013) The PRoteomics IDEntifications (PRIDE) database and associated tools: status in 2013. Nucleic Acids Res. 41, D1063–D1069 23203882 10.1093/nar/gks1262 PMC3531176

[48] Steinbusch M. M. F., Caron M. M. J., Surtel D. A. M., Friedrich F., Lausch E., Pruijn G. J. M., Verhesen W., Schroen B. L. M., van Rhijn L. W., Zabel B., and Welting T. J. M. (2017) Expression of RMRP RNA is regulated in chondrocyte hypertrophy and determines chondrogenic differentiation. Sci. Rep. 7, 6440 10.1038/s41598-017-06809-5 28743979 PMC5527100

