# Supplementary material for: The antiviral protein viperin regulates chondrogenic differentiation via CXCL10 protein secretion
Source: J Biol Chem. 2019 Feb 4;294(13):5121–36. doi: 10.1074/jbc.RA119.007356 (PMC6442052; doi:10.1074/jbc.RA119.007356)
Supplement: Supporting Information [file supp_294_13_5121__index.html]

The anti-viral protein viperin regulates chondrogenic differentiation via CXCL10 protein secretion — Viperin in chondrogenic differentiation — The antiviral protein viperin regulates chondrogenic differentiation via CXCL10 protein secretion — Viperin in chondrogenic differentiation — Supporting Information 

# The antiviral protein viperin regulates chondrogenic differentiation via CXCL10 protein secretion

## Supporting Information

- Supporting Information (to be published online) - Supplementary Figure 1
