# Supplementary material for: The antiviral protein viperin regulates chondrogenic differentiation via CXCL10 protein secretion
Source: J Biol Chem. 2019 Feb 4;294(13):5121–36. doi: 10.1074/jbc.RA119.007356 (PMC6442052; doi:10.1074/jbc.RA119.007356)
Supplement: Supporting Information [file supp_RA119.007356_142856_1_supp_274748_plw0rc.pdf]

SUPPLEMENTARY FIGURE

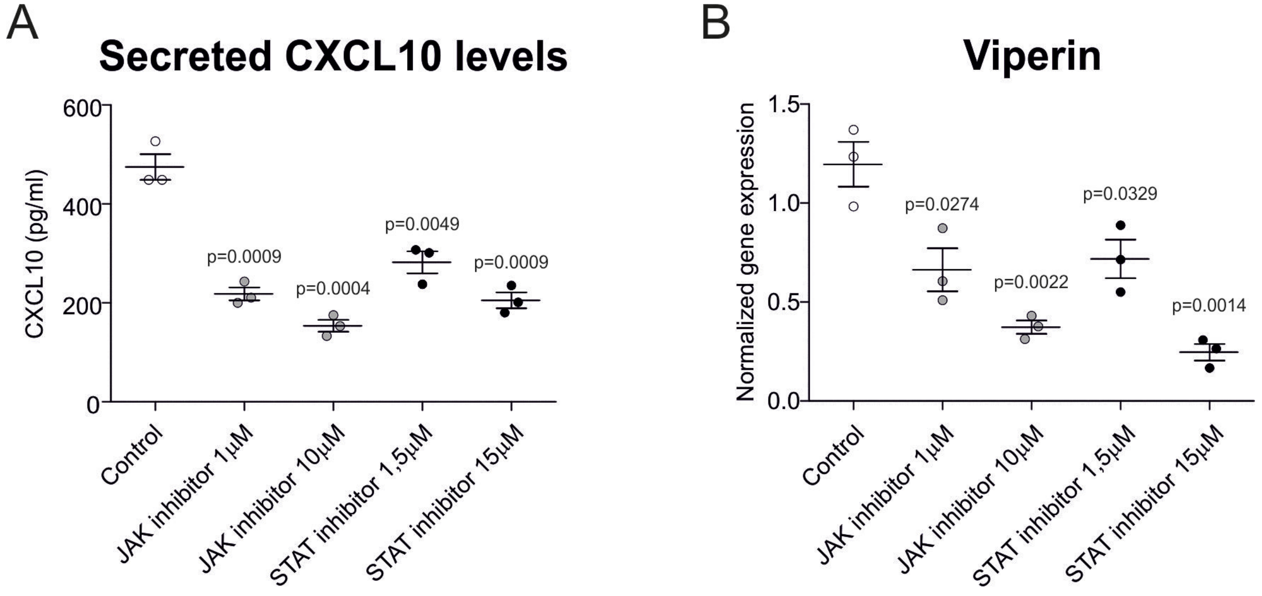

Supplementary Figure 1. Inhibition of interferon signaling activity during ATDC5 chondrogenic differentiation inhibits secreted CXCL10 levels in culture supernant and attenuates viperin expression

ATDC5 cells were differentiated into the chondrogenic lineage until day 6. Downstream interferon signaling activity was then inhibited for 24 hours until day 7. The JAK inhibitor Ruxolitinib was used at 1 μM or 10 μM. The STAT inhibitor Fludarabine was used at 1.5 μM or 15 μM. Control was treatment with vehicle. At day 7 culture supernatant was collected and cells were harvested for total RNA isolation, followed by cDNA synthesis. In culture supernatants secreted CXCL10 protein levels were determined (**A**). Expression of viperin was determined by RT-qPCR Sox9 (**B**). Secreted CXCL10 data are absolute concentrations (pg/mL) and presented in dot plots. RT-qPCR data was normalized to β-actin mRNA levels and individual normalized values are presented in dot plots. All data were acquired from 3 biological replicates. An independent samples t-test was performed relative to control using GraphPad Prism 5. The p-values are indicated and error bars represent mean ± SEM.
